# Anterior shoulder dislocation with avulsion fracture of the greater tuberosity results in reliable good outcomes after closed reduction

**DOI:** 10.1016/j.jseint.2023.12.008

**Published:** 2024-01-17

**Authors:** Gabriele Cirigliano, Franziska Altorfer C.S., Michel Meisterhans, Paul Borbas, Karl Wieser, Florian Grubhofer

**Affiliations:** Department of Orthopedics, University of Zurich, Balgrist University Hospital, Zurich, Switzerland

**Keywords:** Anterior shoulder dislocation, Greater tuberosity fracture, Anterior shoulder fracture dislocation, Conservative treatment, Fracture avulsion greater tuberosity, Greater tuberosity dislocation

## Abstract

**Background:**

Avulsion of the greater tuberosity (GT) due to traumatic anterior shoulder dislocation (ASD) is a commonly observed fracture pattern. After closed reduction of the dislocated humerus, the GT typically reduces itself into its anatomic position enabling the patient to undergo conservative treatment. The aim of this study was to retrospectively review a consecutive series of patients with conservatively treated GT avulsion fractures after closed reduction of an ASD and analyze radiographic outcome, shoulder function and glenohumeral stability and the conversion rate to surgical treatment.

**Methods:**

All patients who underwent closed reduction of a GT avulsion fracture after ASD with the primary intention of conservative treatment between 2017 and 2022 were included. Complications (i.e. conversion to surgical treatment), shoulder function assessed with the American Shoulder and Elbow Surgeons score and subjective shoulder value, instability assessed with the Western Ontario Shoulder Instability score, radiological impingement (greater tuberosity index = GTI and impingement index = II) and GT fracture pattern were assessed as outcome measurements.

**Results:**

A total of 29 patients (mean age 44 years, 27% female) with a mean follow-up of 32.6 (range, 8-96) months were enrolled. Seven patients (24%) underwent surgery due to secondary displacement (n = 4, 14%) or impingement symptoms (n = 3, 10%). All patients who underwent secondary surgery showed a multifragmentary fracture pattern of the GT. Shoulder stiffness (n = 7) and neuropraxia of the axillary nerve (n = 3) were observed temporarily and resolved during the follow-up period. The American Shoulder and Elbow Surgeons and subjective shoulder value of the conservatively treated patients at the last follow-up was 89.2 ± 19.1 respectively 86 ± 18.2%. No recurrent glenohumeral dislocation was documented. The mean Western Ontario Shoulder Instability score at last follow-up was 8(0-71). The mean GTI decreased from 1.2 ± 0.1 after ASD to 1.1 ± 0.1 at the last follow-up (*P* = .002). The mean II decreased from 0.6 ± 0.5 after ASD to 0.4 ± 0.3 at the last follow-up (*P* = .110).

**Conclusion:**

The GT avulsion fragment reduces typically into a close to anatomic position after closed reduction and the GTI even improves with further conservative treatment over time. Close radiological follow-up is necessary to rule out secondary displacement which occurs typically in a multifragmentary fracture pattern. Patients without the need for surgery showed good clinical outcomes without recurrence of glenohumeral instability.

Traumatic shoulder dislocation occurs in up to 23.9/100′000.^12^^,^^26^ Concomitant greater tuberosity (GT) fractures are seen in approximately 10%-30% of these cases.^9^ In the dislocated state of the glenohumeral joint, the degree of GT displacement is typically large. After closed reduction, anatomic or almost anatomic reduction of the GT can usually be observed.^3^ Several studies have shown that persistent displaced GT fractures have a greater risk of subacromial impingement, cuff-rotator-dysfunction, and pseudoarthrosis.^15^^,^^16^^,^^18^^,^^20^^,^^1^ The dislocation of the GT reduces the amount of subacromial clearance as the arm is elevated, leading to limited abduction and external rotation.^15^^,^^16^^,^^18^^,^^1^^,^^20^ The amount of displacement after reduction has been advocated to be a prognostic factor of shoulder function restitution.^1^^,^^8^ Park et al suggested operative treatment for a more than 5 mm displaced GT fracture in young active patients and more than 3 mm in patients involved in overhead activities.^20^ In 2019, Nyffeler et al showed that the greater tuberosity index (GTI) and impingement index should be used to quantify the amount of displacement of GT fractures since it considers the individual anatomical variation of the subacromial space which is created by the anatomy of the humerus with the GT but also the acromion.^17^ According to the current literature, conservative treatment is limited to non- or minimally displaced fractures.^23^ Limited literature exists on the conservative treatment outcomes of dislocated GT fractures after closed glenohumeral reduction following dislocation.^21^^,^^25^

The aim of this study was to retrospectively review a consecutive series of patients with conservatively treated GT avulsion fractures after closed reduction of an anterior shoulder dislocation (ASD) and analyze radiographic outcome, shoulder function and glenohumeral stability and the conversion rate to surgical treatment.

## Material and methods

### Study design and patient selection

This single-center study was performed after institutional review board approval (EK-Nr 2022-02139) was given. A retrospective review was done on all consecutive patients undergoing conservative treatment for isolated GT fracture after ASD, between June 2017 and June 2022. Patients having their closed reduction in a different clinic and starting a conservative treatment at our institution afterwards were also included. Exclusion criteria were: Follow-up of less than 6 months, concomitant fractures of the proximal humerus or the glenoid, previous shoulder instability or previous shoulder operations. Patients had a clinical and radiological follow-up 1, 2, 6, 12 weeks, 6 and possibly 12 months after ASD.

### Intervention: closed reduction and conservative treatment protocol

In the presence of anteroinferior dislocation with GT avulsion, we generally perform fluoroscopic controlled closed reduction and then repeat the radiological examination with X-ray and CT to decide whether surgical fixation of the GT is indicated or not. Closed reduction was performed under general anesthesia and complete muscle relaxation in all cases. Pan et al found that 17 iatrogenic fractures occurred when patients were under moderate sedation, while only 4 cases occurred under general anesthesia. They concluded that maintaining an optimal level of muscle relaxation reduces the risk of iatrogenic fractures.^19^ The risk of an iatrogenic After closed reduction, the conservative treatment protocol consisted of immobilization of the shoulder in internal rotation for 6 weeks in an arm sling. Passive pendulum exercises were performed out of the sling during this period. Free active range of shoulder motion was permitted at week 7 without permission of combined abduction/external rotation until 12 weeks after ASD. Slowly increasing strengthening exercises started after 10 to 12 weeks. Return to contact sport was permitted 16 weeks posttraumatic.

### Clinical outcome parameters

Complications such as conversion to surgical treatment due to secondary displacement of GT, axillary nerve palsy or secondary subacromial impingement were assessed as primary end point. Shoulder function was assessed as secondary end point with collection of subjective shoulder value (SSV) and American Shoulder and Elbow Surgeons (ASES). Patient acceptable symptom state was determined to be an ASES of a minimum of 76.^2^ Shoulder instability was set as third end point with the Western Ontario Shoulder Instability (WOSI) score and redislocation was recorded. The indication for conversion to surgery was made by experienced shoulder surgeons. The operated patients were excluded from the clinical and radiological outcome measurements.

### Radiological outcome parameters

**Figure 1:**
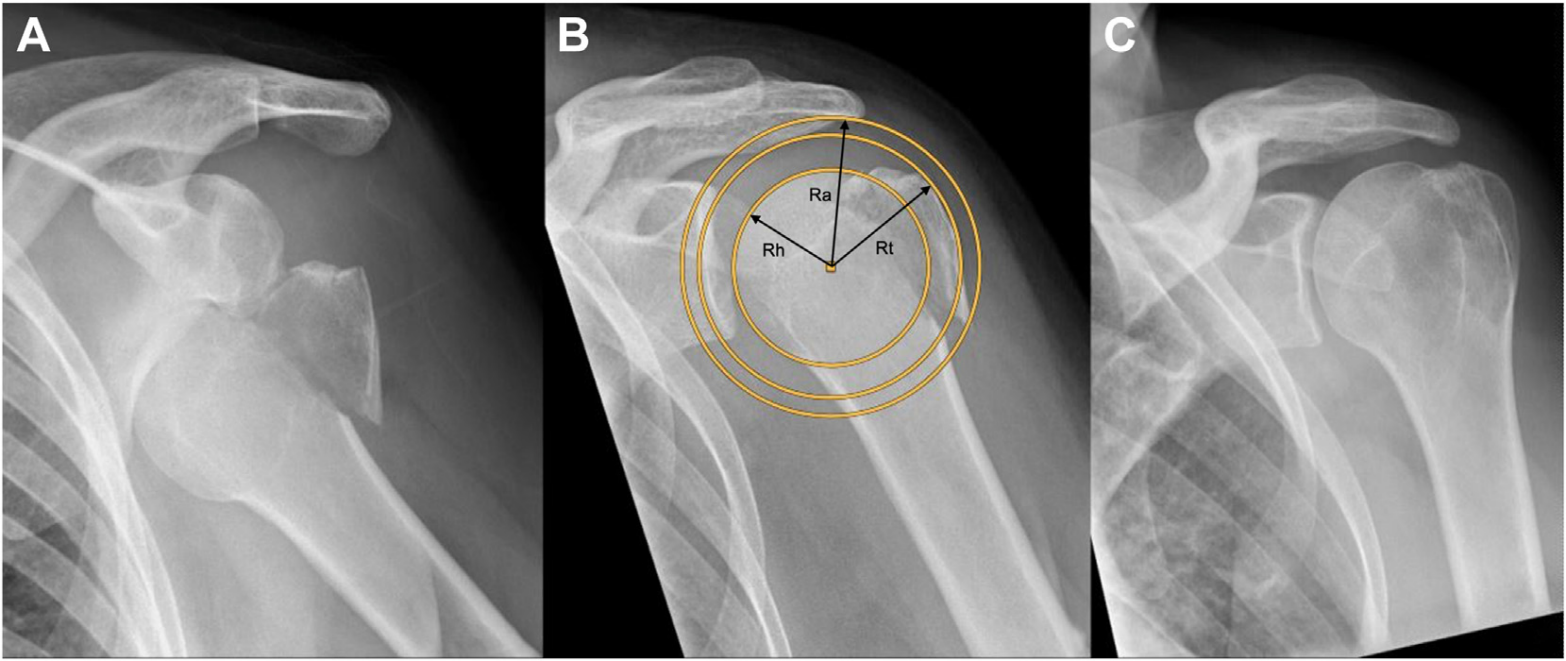
ap radiographs of the glenohumeral joint of two patients showing how the greater tuberosity avulsion fragment after ASD reduces into a close to an anatomic position after closed reduction. From the *Left* to the *Right*: (**A**) radiograph after ASD, (**B**) check-up after closed reduction, (**C**) last follow-up. After closed reduction: GTI: 1.32, II: 1.07. At final follow-up GTI:1.15, II: 0.49. Formula: GTI=Rt/Rh; II=(Rt-Rh)/(Ra-Rh). *ASD*, anterior shoulder dislocation; *GTI*, greater tuberosity index; *II*, impingement index; *Rh*, Radius of a circle tangent to humeral articular surface; *Rt*, Radius of a circle tangent to the greater tuberosity; *Ra*, Radius of a circle tangent to the undersurface of the acromion.

The radiological protocol included a digital upright true anteroposterior radiograph of the glenohumeral joint, and a lateral scapular (y) view.^2^ Humeral head pseudosubluxation (HHPS) that is typically seen in axillary nerve palsy or due to loss of the negative intra-articular glenohumeral pressure or hematoma formation following fracture was measured with humeral head to glenoid ratio described by Dimitriou et al.^5^ Occurrence of radiological impingement was assessed at all scheduled follow-up on standard anteroposterior radiograph with the II and GTI according to Nyffeler et al and was defined as fourth end point.^17^ The subacromial relationships between GT and acromion were calculated using the formula described by Nyffeler et al: Rt/Rh = GTI and (Rt-Rh)/(Ra-Rh)= II (Fig. 1).^16^ Additionally, the fracture pattern (single vs. multifragmentary) was assessed radiologically.^5^ The radiographic measurements were carried out by two independent investigators (orthopedic surgeons) who were not involved in the treatment of the included patients to eliminate bias.

### Statistical analysis

A Shapiro–Wilk normality test and Kolmogorov–Smirnov test were performed to determine if the data were normally distributed. Two-tailed paired *t*-test was used for normally distrusted paired data, whereas Wilcoxon signed rank test was applied for non-normal distributed paired data. Two independent groups with normally and non-normally distributed data were compared using independent T test and Mann–Whitney test, respectively. Continuous variables were described as mean and standard deviation. *P* < .05 was set for statistical significance. The statistical analyses were computed using SPSS version 23 (IBM Corp. Armonk, NY, USA).

## Results

A total of 29 patients with a mean age of 44 ± 16 years met the inclusion criteria. All basic demographic data are summarized in Table I. Twenty-two patients were treated conservatively until the last follow-up with a mean follow-up of 33 (range, 8-96) months. One patient, secondary operated, was lost to clinical follow-up due to lack of current contact information after moving abroad.

**Table I:** Basic demographic.

Demographics	
Patients, number	29
Follow-up, months (range)	33 (8-96)
Lost to follow-up, n (%)	1 (3)
Age, years (SD)	44 (16)
Female, n (%)	8 (27)

*SD*, standard deviation.

### Complications

After an initial conservative treatment, 7 patients (24%) converted to a surgical procedure during follow-up period. Out of these patients, 4 (57%) showed a secondary displacement of the GT and were operated within the first 6 weeks (mean 22 days). In the other 3 (43%) patients arthroscopic subacromial decompression surgery was indicated after 11 months (standard deviation ±6 months) due to subacromial impingement that didn’t respond to conservative treatment (cortisone injection and physiotherapy). In one patient arthroscopic subacromial decompression due to subacromial impingement was indicated and performed in another hospital. A multifragmentary fracture pattern was observed in all 7 secondary operated patients (100%). 6 out of 7 patients were contacted by phone after a mean follow-up of 60 (range, 6-124) months to assess the SSV and ASES which was 73% ± 25 and 65 ± 35.5 respectively. Of the conservative treated patients transient shoulder stiffness (n = 7) and transient axillary nerve injury (n = 3) were observed during the clinical surveillance but didn't impair the clinical outcomes at the last follow-up (Table II).

**Table II:** Complications.

Complications	n (%)
Conversion to surgery	7 (24)
Reason for conversion	
Secondary displacement	4
Subacromial impingement	2
Indicated by external hospital	1
Transient post-traumatic shoulder stiffness	7 (24)
Transient nerve palsy	3 (10)
Instability recurrence	0

### Clinical results

The SSV improved from 9.6% ± 10.3 to 86% ± 17.3 (*P* < .05). The mean ASES was 89.4 ± 19.1 at last follow-up. The Patient acceptable symptom state threshold (ASES, 76 points) was achieved in 91.3% of the patients at the final follow-up. The mean WOSI score at last follow-up was 8 points (range, 0-71) (Table III). No redislocation was observed.

**Table III:** Table showing the SSV at different time points.

Mean (SD)	After reduction	6 weeks after trauma	Last follow-up	*P* value
SSV	9.6 (±10.3)	36.6 (±16.4)	86 (±17.3)	<.001
ASES			89 (±19)	
WOSI			8 (0-71)	

*SSV*, subjective shoulder value; *ASES*, American Shoulder and Elbow Surgeons; *WOSI*, Western Ontario Shoulder Instability score; *ASD*, anterior shoulder dislocation.

The first assessment at 7-10 days after ASD. The second assessment 6 weeks after ASD and the third at the last follow-up. The ASES and WOSI score were assessed at the last follow-up.

### Radiological results

**Figure 2:**
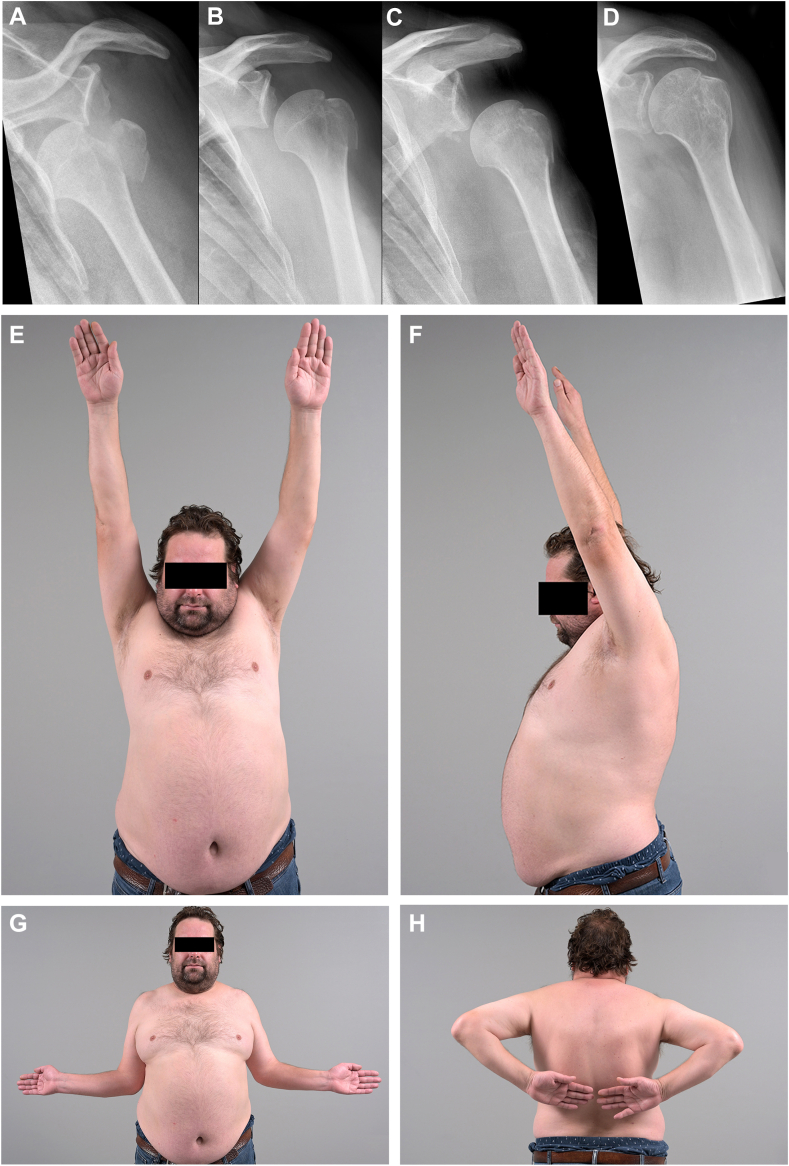
(**A**) ap x ray showing ASD with avulsion fracture of GT. (**B**) ap x ray after closed reduction with reduced GT fragment and HHPS. SSV 0%. (**C**) 6 weeks after ASD and closed reduction with severe HHPS and healed GT fragment. SSV:15%. (**D**) ap x-ray 10 months after ASD and closed reduction showing recentered humeral head and normalized impingment indices. SSV: 99%. (**E-H**) photographic documentation of ROM at last follow-up (10 months). Flexion 160° (**E** and **F**), external rotation 70° (**G**). internal rotation L3 (**H**). *ASD*, anterior shoulder dislocation; *GT*, greater tuberosity; *SSV*, subjective shoulder value; *HHPS*, humeral head pseudosubluxation; *ROM*, range of motion.

The mean GTI decreased from 1.17 ± 0.11 to 1.13 ± 0.11 at 6 weeks, and to 1.09 ± 0.08 at the last follow-up (*P* = .002). The mean II showed likewise a decreasing trend from 0.56 ± 0.53 after reduction, 0.48 ± 0.39 at 6 weeks, and 0.37 ± 0.32 at the final follow-up, although without statistical significance (*P* = .110) (Table IV, Figure 1, Figure 2 and Figure 1, Figure 2). Ten patients (45.5%) showed a typical single avulsion fragment after ASD, while a multiframentary pattern was observed in the remaining 12 patients (54.5%). Patients who underwent conversion to surgery had significantly more often a multifragmentary fracture pattern compared to conservatively treated patients (100% vs. 55%, *P* = .03) - Table V. No statistical difference was observed regarding the fragmentary pattern when compared with GTI and II. A temporary HHPS was observed in the radiographs of 5 patients, clinically remarkable in only 3 of them (Fig. 2). All patients showed complete remission of the humeral head pseudo subluxation at the last follow-up.

**Table IV:** Table showing GTI and II at different time points.

Mean (SD)	After reduction	6 weeks after trauma	Last follow-up	*P* value
GTI	1.17 ± 0.11	1.13 ± 0.11	1.09 ± 0.08	.002
II	0.56 ± 0.53	0.48 ± 0.39	0.37 ± 0.32	.1

*GTI*, greater tuberosity index; *II*, impingement index; *SD*, standard deviation.

The subacromial relationships between GT and acromion were calculated using the formula described by Nyffeler et al.^17^

**Table V:** Table showing the fracture pattern in conservative treated patients as well as in the secondary operated patients.

N° (%)	Conservative n = 22	Conversion n = 7	*P* value
Multifragmentary	12 (54.5%)	7 (100%)	.03
Avulsion	10 (45.5%)	0	

## Discussion

The most important finding of the study was that conservative treatment of GT fractures after shoulder dislocation can lead to good clinical results and good shoulder function at mid-term follow-up, confirmed by a high SSV and ASES score when no secondary displacement of the GT fragment was observed. In our cohort 4 patients out of 29 (14%) showed a secondary GT dislocation within 6 weeks with conversion to surgical treatment. Multifragmentary fracture pattern of the GT could be identified as a risk factor for secondary displacement. Recurrent radiological controls are, therefore, necessary to rule out secondary displacement of the GT.

Recurrent shoulder instability is the most common complication following primary shoulder dislocation, especially among young patients.^14^^,^^10^ We investigated this parameter using the WOSI questionnaire and we found that none of the 29 patients suffered from recurrence of glenohumeral instability in a relatively short follow-up period. There are possible explanations for the observed low risk of recurrence. One plausible theory is that the follow-up time of 33 months with a minimum follow-up of 8 months was too short. Another reason for the low recurrence rate might be the concomitant fracture of the GT that decreases the compression forces within the joint during the dislocation episode, subsequently diminishing the probability of damaging the anterior glenoid rim and anterior capsulolabral structures.^6^ The mean age of the patients is probably another factor influencing the recurrence rate, as it is known that the development of recurrent dislocation is higher in younger patients.^10^^,^^22^^,^^15^

We assessed the radiographs for possible secondary subacromial impingement of the GT using the method suggested by Nyffeler et al, which considers the individually variable width of the subacromial space.^17^ According to this study, an Impingement Index greater than 0.75 and a GTI greater than 1.26 are associated with subacromial impingement. One of the most interesting findings of the present study was to observe a normalization of the impingement indices in the conservative treated patients over time (Fig. 1). Serial radiological follow-up is, therefore, needed to avoid a premature decision to proceed with surgery based on the first “pathological values”. While the clinical outcomes for acute fixation of isolated GT fracture are known, data about delayed surgical intervention are lacking and may differ.^11^ We reported transient shoulder stiffness (24%) and transient axillary nerve injury (10%), whereby neither frozen shoulder nor the temporary nerve damage impaired the clinical outcomes at the last follow-up. According to the literature, peripheral nerve injury occurs in about one-third of GT fracture-dislocation and is most commonly a neuropraxia or low-grade axonotmesis due to the stretch or external pressure of the trauma.^7^^,^^13^^,^^24^ Most of these injuries heal without surgery.^7^^,^^13^^,^^24^ In the same context an injury to the axillary nerve has been theorized to be responsible for deltoid muscle dysfunction and temporary inferior subluxation of the humeral head. A HHPS (HHPS) was observed in the radiographs of 5 patients (21%), which is consistent with the incidence of HHPS found in the literature after acute proximal humerus fractures.^4^ In the present study all 5 patients with HHPS showed spontaneous resolution of the neuropraxia of the axillary nerve and consecutive complete resolution of the HHPS. It is very important to be aware that HHPS is a radiological finding in patients with (transient) axillary nerve palsy and that it is not associated with shoulder instability. If HHPS is misinterpreted as a sign of glenohumeral instability, the surgeon will be misled and might indicate an unnecessary surgery. Therefore, the knowledge of both: HHPS as result of (transient) axillary nerve palsy and low incidence of glenohumeral shoulder instability recurrence after ASD with GT avulsion fracture is crucial to avoid unnecessary surgery. In one case the pseudosubluxation was very advanced but no further intervention was necessary, and the patient showed normal shoulder function with a SSV of 99% at the final follow-up (Fig. 2).

### Limitations

The results of the present study should be interpreted in the context of multiple limitations. First, the sample size is small. Second, the observational rather than comparative nature of the study reduces the quality of the data. Third, the follow-up is limited, and longer study periods are, therefore, required to especially assess recurrence of instability.

## Conclusion

In absence of secondary GT displacement conservative therapy of GT fractures after closed reduction of an ASD is associated with good clinical results and no recurrence of glenohumeral instability. Multifragmentary fracture pattern of the GT fragment is a risk factor for secondary displacement of the GT and close radiological follow-up is, therefore, mandatory. If no secondary displacement occurs the initial degree of displacement of the GT reduces significantly during the course of conservative treatment and allows anatomical restoration of the proximal humerus.

## Disclaimers:

Funding: No funding was disclosed by the authors.

Conflicts of interest: The authors, their immediate families, and any research foundation with which they are affiliated have not received any financial payments or other benefits from any commercial entity related to the subject of this article.
